# A basic investigation into the optimization of cylindrical tubes used as acoustic stethoscopes for auscultation in COVID-19 diagnosisa)

**DOI:** 10.1121/10.0002978

**Published:** 2021-01-05

**Authors:** Chuanyang Jiang, Jiaqi Zhao, Bin Huang, Jian Zhu, Jiao Yu

**Affiliations:** 1 College of Mechanical Engineering, Liaoning Shihua University, Fushun, Liaoning Province, 113001, People's Republic of China; 2 Department of Ultrasound, Changzheng Hospital, Second Military Medical University, Shanghai, 200003, People's Republic of China; 3 The First School of Clinical Medicine, Southern Medical University, Guangzhou, Guangdong Province, 510515, People's Republic of China; 4 Department of Thoracic Cardiovascular Surgery, General Hospital of Central Theater Command of the People's Liberation Army, Wuhan, Hubei Province, 430000, People's Republic of China; 5 College of Science, Liaoning Shihua University, Fushun, Liaoning Province, 113001, People's Republic of China

## Abstract

During the COVID-19 outbreak, the auscultation of heart and lung sounds has played an important role in the comprehensive diagnosis and real-time monitoring of confirmed cases. With clinicians wearing protective clothing in isolation wards, a potato chip tube stethoscope, which is a secure and flexible substitute for a conventional stethoscope, has been used by Chinese medical workers in the first-line treatment of COVID-19. In this study, an optimal design for this simple cylindrical stethoscope is proposed based on the fundamental theory of acoustic waveguides. Analyses of the cutoff frequency, sound power transmission coefficient, and sound wave propagation in the uniform lossless tube provide theoretical guidance for selecting the geometric parameters for this simple cylindrical stethoscope. A basic investigation into the auscultatory performances of the original tube and the optimal tube with proposed dimensions was conducted both in a semi-anechoic chamber and in a quiet laboratory. Both experimental results and front-line doctors' clinical feedback endorse the proposed theoretical optimization.

## I. INTRODUCTION

The COVID-19 pandemic has become an enormous public health concern, attracting intense attention not only in China but also around the globe. Given the high contagiousness of severe acute respiratory syndrome coronavirus 2 (SARS-CoV-2),^1^ strict personal protective measures have been implemented by Chinese medical workers who have worked in infectious isolation wards. In addition, the rigorous use of personal protective equipment, especially protective clothing, makes auscultation with conventional acoustic stethoscopes impossible.^2^ However, it is essential to use stethoscopes to assess the subtleties of the heart and lung sounds and monitor the progression of pneumonia with COVID-19 dynamically.

Under these circumstances, Zhu *et al.*^2^ have reported that a simple, disinfected cylindrical stethoscope made from an empty potato chip tube was applied during the first-line treatment of COVID-19. Although economical and safe, it is essential that this type of stethoscope, which is directly used in auscultation, is subjected to acoustic analysis and designed to obtain a more reliable acoustic performance. Hence, the aim of the present investigation is to provide an optimal design for this alternative stethoscope.

## II. THEORETICAL ANALYSIS

### A. Cutoff frequency analysis

In this study, a potato chip tube is modeled as a cylindrical rigid-walled waveguide with a diameter *d* = 6.53 cm. According to the basic theory of acoustic waveguides in constant cross section,^3^ the cutoff frequency of the plane wave in the hollow cylindrical waveguide is

$$f_{c}=\frac{1.841c_{0}}{2\pi a}=\frac{1.841\times 344}{\pi \times 6.53\times 10^{-2}}=3087.1\;\;\text{Hz},$$ (1)

where a is the radius of the circular cross section and $c_{0}$ is the speed of sound in air at 20 °C. Considering that all of the physiologically crucial cardiopulmonary sounds^4,5^ are evidently less than the $f_{c}$ in Eq. (1), the sound used for auscultation will propagate only in the plane-wave mode. Therefore, this pure propagation mode can greatly assist physicians and, particularly, cardiologists in dynamically monitoring as well as making accurate judgments regarding individual cardiopulmonary function.

### B. Diameter selection analysis of the cylindrical stethoscope

**FIG. 1.**
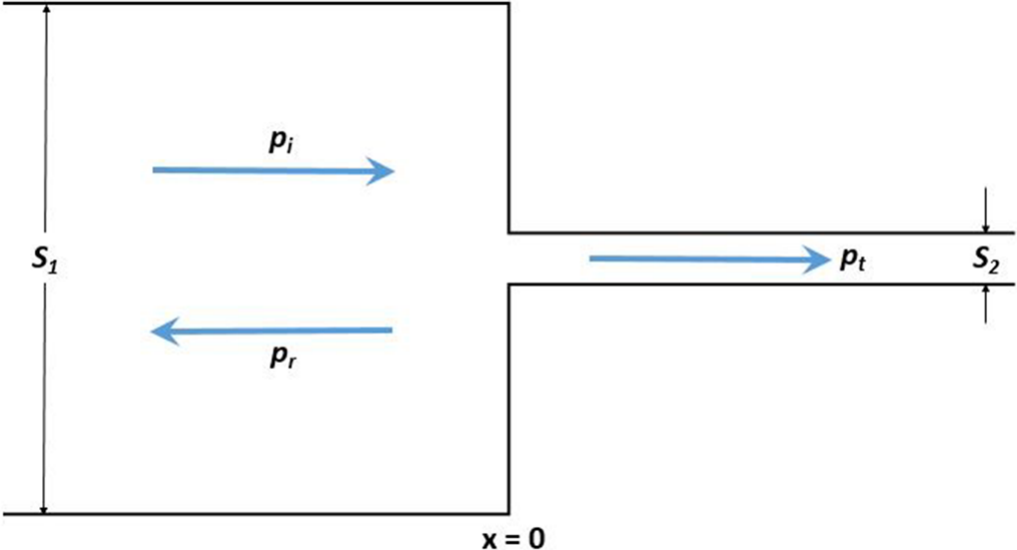
(Color online) Transmission and reflection of planar sound waves in the vicinity of a junction between two waveguides where the cross-sectional area changes from $S_{1}$ to $S_{2}$.

In addition to meeting the cutoff frequency requirements, sound transmission to the external auditory canal must be considered in the design. To explore the essence of this research, slight differences in external auditory canal shapes and diameters between individuals are ignored. Standing waves do not need to be considered because such patterns usually occur at frequencies above 6000 Hz in the human auditory canal.^6–8^ Within the scope of the above approximation, the acoustic model that describes a circular waveguide with a sudden change in the cross section is illustrated in Fig. 1 to analyze the sound transmission during auscultation.

It is assumed that the coordinate origin is precisely at the junction between these two waveguides that represent the simple cylindrical stethoscope of the cross-sectional area $S_{1}$ and a physician's external auditory canal of the cross-sectional area $S_{2}$. It should be noted that the boundary conditions must include continuity in the pressure and volume velocity. As a result, the sound power transmission coefficient in the simplified acoustic duct of the varied cross section^9^ is

$$t_{w}=\frac{4\left(\frac{S_{1}}{S_{2}}\right)}{{\left(\frac{S_{1}}{S_{2}}+1\right)}^{2}}.$$ (2)

**FIG. 2.**
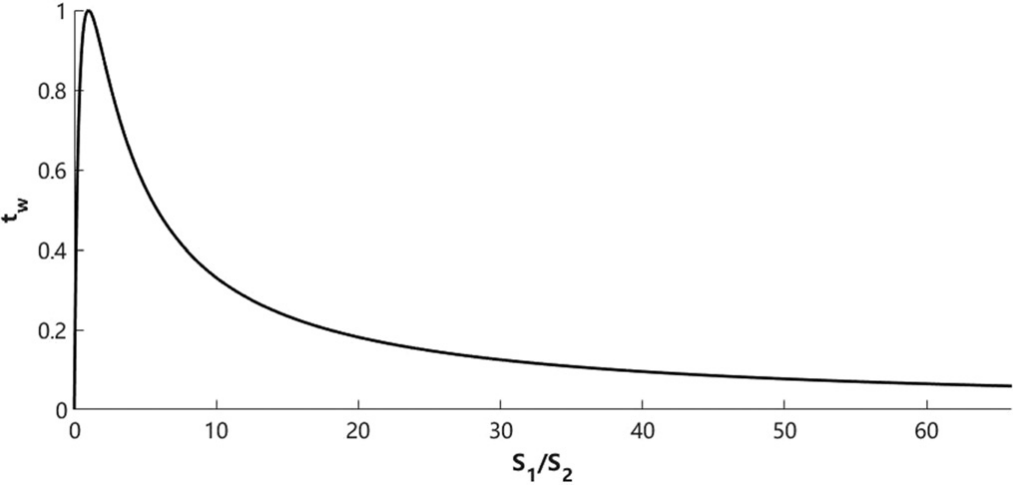
The relationship between the sound power transmission coefficient and the area ratio of $\;S_{1}$/$S_{2}$.

The relationship between $t_{w}$ and $S_{1}/S_{2}$ is plotted in Fig. 2, which indicates that the sound power transmission coefficient reaches the maximum when $S_{1}$ equals $S_{2}$ in this application.

In a real clinical situation, the size of the chest piece of the acoustic stethoscope, regardless of the diaphragms or the bells, is not as small as that of the cross section of the external auditory canal (typically approximately 0.8 cm in diameter^10^) but it ranges from approximately 3.3 to 4.4 cm^11^ due to some factors that influence the picking up sounds and vibration, including the effective auscultation area of heart and lung sounds, as well as the curvature of the human body surface.

### C. Length selection analysis of the cylindrical stethoscope

For the optimal length of the cylindrical stethoscope, the fundamental equations that describe the sound wave propagation in the uniform, lossless waveguide are^12^

$$-\frac{\partial p}{\partial x}=\frac{\rho }{A}\frac{\partial u}{\partial t},$$ (3)

$$-\frac{\partial u}{\partial x}=\frac{A}{\rho c^{2}}\frac{\partial p}{\partial t},$$ (4)

where p is the sound pressure, ρ is the density of air in the waveguide, *A* is the cross-sectional area, c is the speed of sound in air, and *u* is the volume velocity at (*x*,*t*). In addition, the boundary conditions are

$$x=0,\;\;\;\;u\left(0,t\right)=U\left(\Omega \right)e^{j\Omega t},$$ (5)

x=l,    p(l,t)=0. (6)

The solution form of u(x,t) can be obtained:

$$u\left(x,t\right)=\frac{\;\text{cos}\;\left[\Omega \left(\frac{l-x}{c}\right)\right]}{\;\text{cos}\;\left(\frac{\Omega l}{c}\right)}U\left(\Omega \right)e^{j\Omega t}.$$ (7)

The Laplace transform of u(l,t)/u(0,t), namely, the transfer function of the cylindrical acoustic waveguide,^12,13^ is

$$Vs=\frac{u\left(l,s\right)}{u\left(0,s\right)}=\frac{1}{\;\text{cos}\;\left(\frac{sl}{jc}\right)}=\frac{2e^{-sl/c}}{1+e^{-s2l/c}},$$ (8)

where s=jΩ (Ω is the angular frequency). To calculate the formant frequency, we let the denominator be zero and reject the negative value of s; thus, we have

$$f=\frac{\Omega }{2\pi }=\frac{s}{2\pi j}=\frac{\left(2n+1\right)c}{4l}\;\;\;\;\left(n=0,1,2,\ldots \right).$$ (9)

From Ref. 2, the length of the commercially available potato chip tube ranges from 20.0 to 30.0 cm. When *l* = 20 cm, the fundamental frequency is

$$f_{0}=\frac{344}{4\times 0.2}\;\;\text{Hz}=430\;\;\text{Hz}.$$ (10)

Clearly, $f_{0}=430\;\;\text{Hz}$ is greater than the frequency range of auscultation at the apex area of the heart, ranging from 20 to 115 Hz.^14^ If the fundamental frequency is 115 Hz, then the corresponding tube length will be 75 cm, which is difficult to use in clinical auscultation. Therefore, for the sake of convenience and availability, it is proper to select the length of the tube at approximately 20 cm.

## III. EXPERIMENTAL TEST AND EVALUATION

According to the theoretical analysis, four volunteers of different sexes, ages, and weights were tested both in a semi-anechoic chamber and in a quiet laboratory. All four volunteers were tested in a calm state, and relevant information, including body mass index, is provided.^15^

The setup consisted of an original potato chip tube stethoscope (diameter, 6.53 cm; length, 20.0 cm), an optimal tube stethoscope (diameter, 4.20 cm; length, 20.0 cm),^15^ a sound level meter (type-2250) with a prepolarized microphone (type-4189) and a microphone preamplifier (ZC-0032), and BK Connect 2019 software (Brüel and Kjær, Nærum, Denmark). In the test, an *A*-weighting sound pressure level and 1/3 octave are used. Also, considering the frequency range of auscultation at the apex area of the heart, we set the lower band as 20 Hz and the higher band as 250 Hz for sound calculations and plots.

In the experimental investigation, each of the four volunteers was tested twice continuously, and each cardiac auscultation at the apex area of the heart lasted for one minute. The mean sound pressure level for each kind of tube is obtained by taking the average of all eight measurements. Considering that medical practitioners need to keep their ears close to the cylindrical stethoscope to obtain clear signals and prevent sound leaks and ambient noise interference, a sponge rubber sheath was employed for sound insulation of the experimental environment. Moreover, to truly reflect the situation of heart auscultation in which the medical staff wears protective clothing, a small piece of material cut from a suit of protective clothing was used for wrapping the front end of the microphone, whose body was inserted into the sponge rubber sheath.^15^ All the described tests were carried out using identical hardware setups and settings.

## IV. RESULTS AND DISCUSSION

**FIG. 3.**
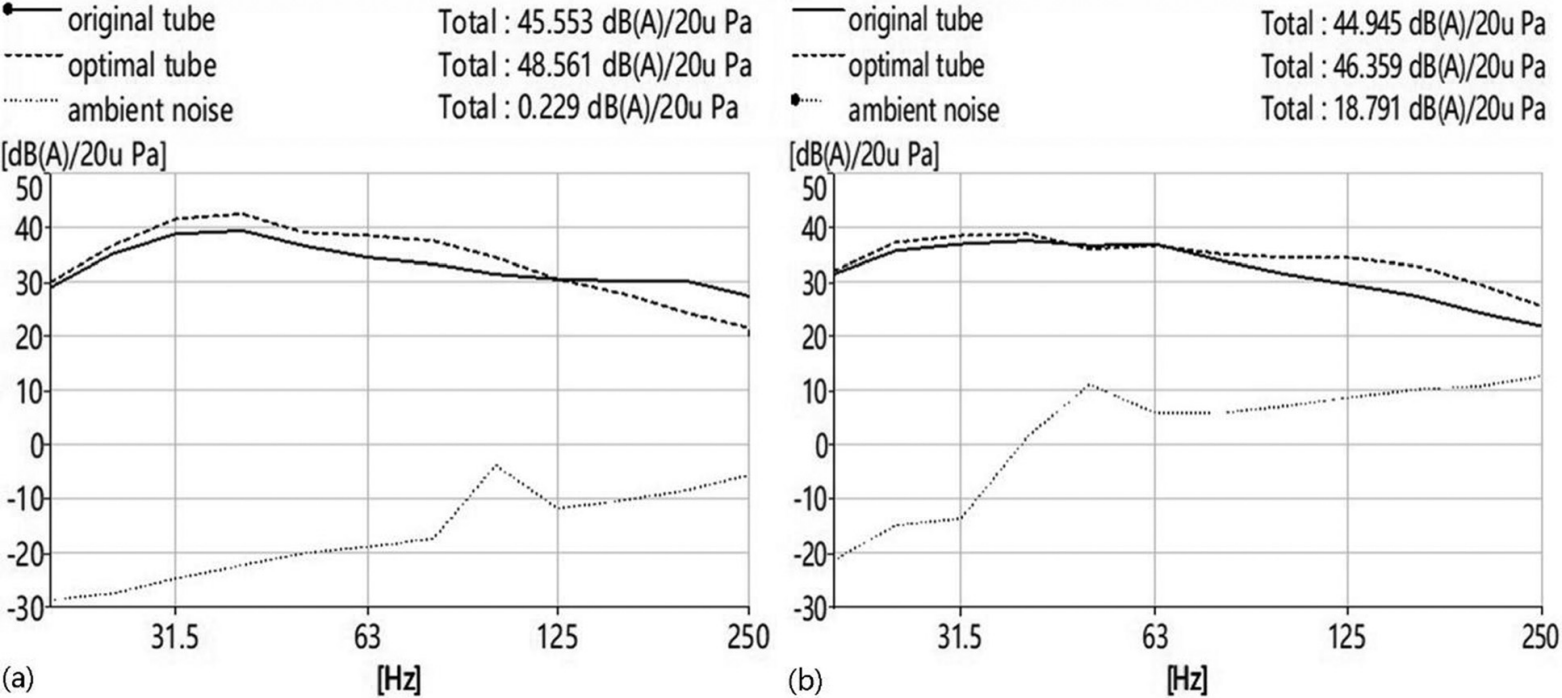
The frequency spectra of sound pressure levels measured by a sound level meter when auscultating at the apex area of the heart. (a) Measured in a semi-anechoic chamber. (b) Measured in a quiet laboratory.

The results of the performed experimental investigations are presented in Fig. 3. It is shown that both the original potato chip tube stethoscope and the optimal tube stethoscope can be utilized for heart auscultation because the measured sound pressure levels of both tubes are distinctively greater than that of the ambient noise. The sound pressure level of the optimal tube stethoscope is approximately 3 dB higher than that of the original potato chip tube stethoscope.

Based on our clinical experience, the auscultatory performance of the optimal tube stethoscope is moderately better than that of the original potato chip tube stethoscope in heart auscultation, and the optimal tube stethoscope, with a diameter of about 4 cm and length of about 20 cm, is feasible in terms of auditory quality, convenience of auscultation, and material availability, as well as a low cost of manufacturing. According to the analysis and evaluation above, we can summarize the basic findings on the optimal design of this simple cylindrical stethoscope. First, the cutoff frequency analysis suggests that the heart sound within the auscultation frequency range will transmit in plane-wave mode within the cylindrical tube stethoscopes herein. Second, compared with the original tube stethoscope (diameter, 6.53 cm), a hollow cylindrical stethoscope with a diameter of 3.3–4.4 cm is believed to possess better auscultatory performance, which has been experimentally and clinically confirmed by using the optimal tube stethoscope (diameter, 4.20 cm). This is partly because the optimized size is suitable for picking up heart sounds and partly because the optimization makes the difference between $S_{1}$ and $S_{2}$ smaller, which also contributes to sound transmission to the external auditory canal. Third, considering auscultation convenience and material availability, a tube length of 20 cm is appropriate. To obtain a fundamental frequency of the formant at 115 Hz or lower, the corresponding tube length needs to be at least 75 cm, which is impractical for device operation.

Moreover, instead of using the original material from the inner wall of the cylindrical stethoscope, which is to some degree infiltrated by oil, an appropriate substitute, such as a coat of enamel, should be painted smoothly and uniformly on the surface to reduce the frictional loss of sound due to viscosity. In addition, a great deal of attention must be paid to the fact that medical workers who intend to conduct auscultations using this simple device should wear a single earplug in the ear that is not being used during auscultation to reduce the impact of ambient noise during diagnosis.

Dating back to 1816, the French doctor Laennec, who invented the world's first stethoscope, successfully conducted a cardiac auscultation using a cylindrical paper stethoscope.^16,17^ After continuous optimization and improvement, modern stethoscopes now possess better acoustic performance during cardiac and pulmonary auscultation. Nevertheless, strict personal protective equipment use makes auscultation using modern stethoscopes impossible in diagnosing confirmed COVID-19 cases. Given the pressing situation, simple cylindrical stethoscopes, which have advantages in the real-time monitoring of patients, motivate us to reflect on reductionism, as well as pragmatism, in the design and use of diagnostic tools during the COVID-19 pandemic.

## V. CONCLUSIONS

In conclusion, during the COVID-19 pandemic, simple cylindrical stethoscopes have been applied to cardiopulmonary function auscultation in China, and this economical instrument is considered a suitable diagnostic tool when conventional stethoscopes cannot be used by medical workers in protective clothing or when medical resources are extremely scarce in poverty-stricken areas of developing countries. Based on the theoretical analyses, experimental results, and doctors' clinical practice, the acoustic performances of both cylindrical tube stethoscopes can be accepted with the optimal tube performing moderately better. Compared with the potato chip tube stethoscope, we propose an optimal design by reducing the diameter from 6.53 cm to approximately 4.20 cm and selecting a tube length of 20 cm, which improves auscultatory quality, convenience of device operation, and economic viability.
